# Search of Potential Vaccine Candidates against *Trueperella pyogenes* Infections through Proteomic and Bioinformatic Analysis

**DOI:** 10.3390/vaccines8020314

**Published:** 2020-06-17

**Authors:** Ángela Galán-Relaño, Lidia Gómez-Gascón, Antonio Rodríguez-Franco, Inmaculada Luque, Belén Huerta, Carmen Tarradas, Manuel J. Rodríguez-Ortega

**Affiliations:** 1 Departamento de Sanidad Animal, Universidad de Córdoba; Campus de Excelencia Internacional CeiA3, 14071 Córdoba, Spain; agalanr12@gmail.com (Á.G.-R.); sa1lumoi@uco.es (I.L.); sa2hulob@uco.es (B.H.); sa1taigc@uco.es (C.T.); 2 Departamento de Bioquímica y Biología Molecular, Universidad de Córdoba, and Campus de Excelencia Internacional CeiA3, 14071 Córdoba, Spain; arfranco@uco.es (A.R.-F.); mjrodriguez@uco.es (M.J.R.-O.)

**Keywords:** *T. pyogenes*, proteomics, pan-surfome, surface proteins, antigens, vaccine

## Abstract

*Trueperella pyogenes* is an opportunistic pathogen, responsible for important infections in pigs and significant economic losses in swine production. To date, there are no available commercial vaccines to control diseases caused by this bacterium. In this work, we performed a comparative proteomic analysis of 15 *T. pyogenes* clinical isolates, by “shaving” live cells, followed by LC-MS/MS, aiming at the identification of the whole set of surface proteins (i.e., the “pan-surfome”) as a source of antigens to be tested in further studies as putative vaccine candidates, or used in diagnostic tools. A total of 140 surface proteins were detected, comprising 25 cell wall proteins, 10 secreted proteins, 23 lipoproteins and 82 membrane proteins. After describing the “pan-surfome”, the identified proteins were ranked in three different groups based on the following criteria: to be (i) surface-exposed, (ii) highly conserved and (iii) widely distributed among different isolates. Two cell wall proteins, three lipoproteins, four secreted and seven membrane proteins were identified in more than 70% of the studied strains, were highly expressed and highly conserved. These proteins are potential candidates, alone or in combination, to obtain effective vaccines against *T. pyogenes* or to be used in the diagnosis of this pathogen.

## 1. Introduction

*Trueperella pyogenes* is a Gram-positive bacterium that is part of the normal biota of skin and mucous membranes of upper respiratory, gastrointestinal, reproductive and urinary tracts of domestic and wild life animals [1]. However, it can be an opportunistic pathogen responsible for purulent infections, such as metritis, mastitis, pneumonia and abscesses, of special importance in livestock breeding animals because of the economic losses it generates [2]. Antimicrobial treatment is the main tool to control the infections caused by this microorganism so far [3]. However, the growing concern about the use of antimicrobials requires studying other alternatives for the control of the diseases caused by this pathogen [4]. Among them, vaccination is one of the most recommended measures, and should be considered a method of first choice to prevent *T. pyogenes* diseases [2]. Various approaches to stimulate a protective immunity against *T. pyogenes* infection in animals have been tried. Whole-cell vaccines based on killed or attenuated strains or culture supernatant have given inconsistent results [5,6,7].

Currently, the focus has shifted towards proteins as vaccine candidate agents, mainly targeting surface proteins, because they are more exposed and thus, more accessible to antibodies. In Gram-positive bacteria, surface proteins are generally grouped into different categories: cell wall anchored proteins (either covalently linked to the peptidoglycan cell wall or bound via weak interactions), membrane proteins (including the integral membrane proteins and lipoproteins, i.e., those anchored to the membrane via an *N*-term linked lipid), and secreted proteins that attach to the surface after being exported to the extracellular milieu [8]. Usually, proteins anchored to the cell wall, lipoproteins and those secreted, are more exposed than transmembrane proteins and thus, more accessible to antibodies. Therefore, they have the best chances to raise a high and effective immune response [9,10,11].

Proteomics tools have been applied for the discovery of new antigens as putative vaccines [12,13]. Recently, several surface proteins have been tested as vaccine candidates for the control of infectious diseases, and some of them have shown protective effect [14,15]. In *T. pyogenes* a recombinant protein, based on the secreted protein pyolysin (PLO), has been tested as a possible vaccine candidate, showing promising results in preliminary studies using murine models of infection [16,17]. However, no other proteins have been proposed as targets for the development of *T. pyogenes* vaccines [18]. Moreover, in order to get a broad cross-protection against multiple isolates, it is essential to analyze a collection of such isolates to identify novel vaccine antigens that are common to all or the majority of the strains [19].

During the last few years, the “surfome” analysis by means of the “shaving” method, consisting of treating live cells with proteases and analyzing the resulting peptides by LC-MS/MS, has demonstrated its power to identify, in a fast and reliable way, tens of surface proteins in a sample [20]. This approach has been used in a lot of bacterial species, allowing researchers to discover new immunoprotective proteins [12,19,21,22].

In this study, we performed for the first time a proteomic analysis of a collection of clinical isolates of *T. pyogenes*, by applying the “shaving” approach. The objective of this work was to describe the “pan-surfome” of this pathogen, i.e., the set of proteins in a wide collection of clinical isolates that can represent the proteome of the species, and to identify surface proteins to propose different antigenic candidates that could be used in further immunization and/or vaccination studies for the development of new vaccines or diagnostic tools.

## 2. Materials and Methods

### 2.1. Bacterial Strains and Culture Conditions

Fifteen *T. pyogenes* isolates recovered from pigs totally or partially condemned at the slaughterhouse after veterinary inspection (Regulation 2004/854/EC) were studied. Those isolates were genetically characterized by our group in a previous work [4]. Samples were obtained from different locations with macroscopic lesions of pneumonia, endocarditis, arthritis, lymphadenitis, abscess or pyogranuloma-like lesions (Table 1). All strains, maintained at −80 °C, were plated on Columbia CNA agar (Oxoid ltd., Hampshire, UK), supplemented with 5% (*v*/*v*) sterile defibrinated sheep blood. Plates were incubated under microaerophilic conditions (5% CO_2_) at 37 °C for 24–48 h [23]. Once grown, the whole bacterial growth was inoculated in 45 mL of brain heart infusion (BHI, Oxoid ltd., Hampshire, UK) [24], and incubated at 37 °C for 48 h under aerophilic conditions. After this incubation, *T. pyogenes* reached an OD_595_ of 0.4, corresponding to mid-exponential phase.

### 2.2. “Shaving” of Bacterial Live Cells and Peptide Extraction

Bacteria from 45 mL cultures at mid-exponential growth phase (approximately 10^7^ cells at OD_595_ = 0.4) were harvested by centrifugation at 3500× *g* for 10 min at 4 °C and washed three times with 20 mL PBS. Cells were resuspended in 0.4 mL of PBS/30% sucrose in a 1.5 mL tube. Proteolytic reactions were carried out with trypsin (Promega, Madison, WI, USA) at 5 μg/mL, for 30 min at 37 °C with top-down agitation. The digestion mixtures were centrifuged at 3500× *g* for 10 min at 4 °C, and the supernatants (the “surfomes” containing the peptides) were filtered using 0.22-µm pore-size filters (Millipore, Burlington, MA, USA). “Surfomes” were re-digested with 2 μg trypsin during 2 h at 37 °C with top-down agitation. Salts were removed using Oasis HLB extraction cartridges (Waters, Milford, MA, USA). Peptides were eluted with increasing concentrations of acetonitrile/0.1% formic acid, according to manufacturer’s instructions. Peptide fractions were concentrated with a vacuum concentrator (Eppendorf, Hamburg, Germany), and kept at −20 °C until further analysis.

### 2.3. Liquid Chromatography-Mass Spectrometry (LC-MS/MS) Analysis

Peptide separation was performed by nano-LC using a Dionex Ultimate 3000 nano UPLC (Thermo Scientific, San Jose, CA, USA), equipped with a reverse phase C 18 75 μm × 50 mm Acclaim Pepmap column (Thermo Scientific) at 300 nL/min and 40 °C for a total run time of 85 min. The mix of peptides was previously concentrated and cleaned up on a 300 μm × 5 mm Acclaim Pepmap cartridge (Thermo Scientific) in 2% acetonitrile/0.05% formic acid for 5 min, with a flow of 5 µL/min. Buffer A (0.1% formic acid) and Buffer B (80% acetonitrile, 0.1% formic acid) were used as a mobile phase for the chromatographic separation, according to the following elution conditions: 4–35% Buffer B for 60 min; 35–55% Buffer B for 3 min; 55–90% Buffer B for 3 min, followed by 8 min washing with 90% Buffer B, and re-equilibration during 12 min with 4% Buffer B.

Peptide positive ions eluted from the column were ionized by a nano-electrospray ionization source, and analyzed in positive mode on a trihybrid Thermo Orbitrap Fusion (Thermo Scientific) mass spectrometer operating in Top30 Data Dependent Acquisition mode, with a maximum cycle time of 3 s. MS1 scans of peptide precursors were acquired in a 400–1500 *m*/*z* range at 120,000 resolution (at 200 *m*/*z*), with a 4 × 10^5^ ion count target threshold. For MS/MS, precursor ions were previously isolated in the quadrupole at 1.2 Da, and then CID-fragmented in the ion trap with 35% normalized collision energy. Monoisotopic precursor selection was turned on. Ion trap parameters were: (i) the automatic gain control was 2 × 10^3^; (ii) the maximum injection time was 300 ms; and (iii) only those precursors with charge state 2–5 were sampled for MS/MS. In order to avoid redundant fragmentations a dynamic exclusion time was set to 15 s with a 10-ppm tolerance around the selected precursor and its isotopes.

### 2.4. Database Searching and Protein Identification

The mass spectrometry raw data were processed using Proteome Discoverer (version 2.1.0.81, Thermo Scientific). Charge state deconvolution and deisotoping were not performed. MS/MS spectra were searched with SEQUEST engine against a database of Uniprot_Trueperella pyogenes_Jun2018 (www.uniprot.org, Taxonomy ID: 1661) containing all the strain sequences available to date, and applying the following search parameters: trypsin digestion with 4 missed cleavages. Methionine oxidation was set as variable modification. A value of 10 ppm was set for mass tolerance of precursor ions, and 0.1 Da tolerance for product ions. Peptide identifications were accepted if they exceeded the filter parameter Xcorr score versus charge state with SequestNode Probability Score (+1 = 1.5, +2 = 2.0, +3 = 2.25, +4 = 2.5). All the identifications were manually inspected to eliminate protein redundancies using BLASTp. For those hits which resulted in homology, that with the highest score was selected, and the other ones were discarded. The mass spectrometry raw data have been deposited to PeptideAtlas (www.peptideatlas.org) with the dataset identifier PASS01586.

### 2.5. Bioinformatic Analysis of Protein Sequences

**Figure 1:**
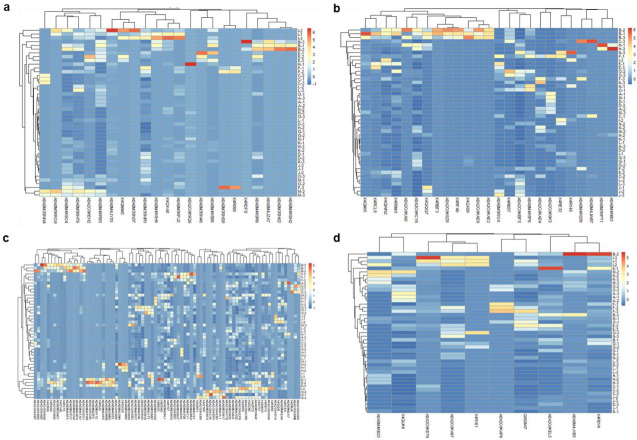
Hierarchically-clustered heatmaps of z-scored surface protein abundances in the 15 *Trueperella pyogenes* clinical isolates. Proteins are clustered in columns in each heatmap, and isolates in rows. The numbers after the dashes in clinical isolates represent each of the three biological replicates. (**a**) Lipoproteins; (**b**) cell wall proteins; (**c**) membrane proteins; (**d**) secreted proteins.

Primary computational predictions of subcellular localization were carried out by using PsortB v3.0 (https://www.psort.org/psortb/). Feature-based algorithms were also used to contrast PsortB predictions: TMHMM 2.0 (http://www.cbs.dtu.dk/services/TMHMM/) for searching transmembrane helices; SignalP 5.0 (http://www.cbs.dtu.dk/services/SignalP/) for type-I signal peptides: those proteins containing only a cleavable type-I signal peptide as the featured sequence were classed as secreted; and LipoP (http://www.cbs.dtu.dk/services/LipoP/) for identifying type-II signal peptides, which are characteristic of lipoproteins. Topological representations of membrane proteins (Figure 1) were performed with the web-based TOPO2 software (http://www.sacs.ucsf.edu/TOPO2/). Moreover, the algorithm VaxiJen (http://www.ddg-pharmfac.net/vaxijen/VaxiJen/VaxiJen.html), based on protein physicochemical properties, was used to predict in silico the protective capacity of the proteins included in the ranking. The VaxiJen model used was “bacterial”, with the threshold fixed on 0.5 [25,26].

### 2.6. Data Analysis and Statistics

For the 15 strains the “shaving” experiments were conducted in triplicate, with each replicate being an independent culture. Proteins were considered to be present in a given sample whenever they were detected in at least two out of the three biological replicates for each strain. Otherwise, proteins found only in one biological replicate were discarded from the overall count of identified proteins for a given strain. For further quantitative analysis, means and standard deviations were calculated using an Excel spreadsheet (Microsoft Excel 2011 v14.0.0 for Mac, Microsoft, Redmond, WA, USA). Z-scored values were calculated before the principal component and clustering analysis was performed. Principal component analysis was done using the R FactoMineR package. The factoextra package was used to represent these analyses, and the pheatmap package was used to cluster the data and represent the corresponding heatmaps. Non-detected proteins in samples were assigned a 0 value to avoid the processing of NA (not available) data.

## 3. Results

### 3.1. Describing the “Pan-Surfome” of T. pyogenes

For this study, the surface proteome of 15 clinical isolates (Supplementary Materials Table S1) was obtained by “shaving” bacterial live cells with trypsin and further LC-MS/MS analysis. We defined the “surfome” of each isolate as the set of predicted surface proteins identified in at least two biological replicates of the given isolate, and the global “pan-surfome” as the set of all the proteins found in the whole collection of strains.

A total of 140 surface proteins were identified in the 15 *T. pyogenes* isolates analyzed, grouped in the following categories (Table 2): 25 were cell wall proteins (representing 17.9% of total identified surface proteins); 10 (7.1%) were proteins possessing a signal peptide I, i.e., proteins secreted via the SPI secretory pathway; 23 (16.4%) were lipoproteins with a signal peptide II; and 82 (58.6%) were membrane proteins with one or more transmembrane domains (TMD). Table 2 also shows the range of proteins of each category detected per isolate.

Interestingly, 11 out of the 25 identified proteins predicted to be cell wall-attached, possessed an LAXTG sortase E-recognizing motif instead of the most common LPXTG, and the membrane protein sortase E was also identified. In addition, 10 of the identified cell wall proteins had an LSXTG motif (Supplementary Materials Table S1).

On the other hand, 820 proteins were classified as cytoplasmic proteins, and 64 proteins were predicted by PsortB to be “unknown”. For these, no exporting motif was identified after manual inspection and using other primary prediction algorithms.

The membrane proteins were those exhibiting the highest expression frequencies: 31 proteins were found at least in the 50% of the analyzed isolates (37.8% of the membrane proteins found in the “pan-surfome”). In a second place, eight cell wall anchored proteins were identified in a minimum of 50% of the isolates (32% of the cell wall proteins described in the “pan-surfome”). Regarding the lipoproteins and secreted proteins categories, six (26.1%) and six (60%) proteins were found in or more than a half of the *T. pyogenes* strains, respectively. However, if we compare the identification frequencies of the different categories in relative terms, the secretory proteins and the cell wall category were the most prevalent ones, as these categories showed the highest number of proteins identified in a high proportion of isolates: six out of 10 proteins (60%) and eight out of 25 proteins (32%) in ≥50% of the isolates, respectively.

### 3.2. Analysis of Differences in Surface Protein Abundances of T. pyogenes Clinical Isolates

After protein identification, we determined the differences in the abundances of surface proteins within the 15 *T. pyogenes* isolates by a label free-based semi-quantitative analysis, based on chromatography peak areas (Supplementary Materials Table S1). First, we performed a principal component analysis (PCA) to evaluate differences in the overall pattern of surface protein abundance when the 15 isolates were compared (Supplementary Materials Figure S1). The measurement of the Euclidean distances showed that in general terms, the three replicates of each isolate were grouped, with some major dispersions in relative terms for isolates A and M, and major absolute dispersions for isolates B, E, and I (Supplementary Materials Figure S2). However, B and E strains were clearly separated from the rest in the principal component (PC) 1 axis, as well as isolate I to a lesser extent, thus indicating that their overall surface protein pattern was different in terms of protein abundances.

Then, we represented in hierarchically-clustered heatmaps the z-scored abundances of the 140 identified surface proteins, grouped according to their subcellular localization: lipoproteins, cell wall, membrane, and secreted proteins (Figure 1). Regarding lipoproteins, the isolate I differed from the others in a relatively higher expression of almost half of the identified lipoproteins, followed by the isolate B to a lesser extent (Figure 1a). Additionally, isolate B was the one showing the highest abundances of many cell wall proteins (Figure 1b). A high diversity and variability of membrane protein abundances was found throughout all the isolates (Figure 1c). However, differences for secreted proteins were less clear (Figure 1d). Especially in these last two categories, there was a greater dispersion of values between replicates. In summary, it appeared that the discrimination in surface protein abundances among isolates was mainly due to lipoproteins and cell wall proteins (at least, regarding separation of B and I from the rest of isolates), and to membrane proteins to a lesser extent.

### 3.3. Ranking of Proteins from the “Pan-Surfome” of T. pyogenes Based on Their Potential as Putative Vaccine Candidates

Finally, the identified surface proteins were ranked in three groups (A, B and C, from best to worst) of a priori potentiality for further immunization and/or vaccination studies on the basis of previous works [16,25,26,27] (Table 3). Briefly, the proteins were ranked according to the following parameters: to be surface expressed, highly conserved and widely distributed among isolates. To ensure the wide distribution of selected proteins among the different isolates, proteins were classified in the three groups: proteins present in more than 70% of the isolates were included in the group A (*n* = 16), proteins present in 50–70% of strains in the group B (*n* = 9), and in the group C, proteins present in 30–50% of strains (*n* = 15).

A special mention is needed for membrane proteins. They are the most embedded ones in the membrane because they have transmembrane domains (TMD). They can be subdivided into membrane proteins with one TMD, which usually have domains in the extracellular side with hundreds of amino acid residues, and membrane proteins with more than one TMD, with a lower probability of having loops large enough to reach the surface and be accessible to antibodies. For this reason, membrane proteins were divided into two sub-groups: membrane proteins with one TMD (*n* = 8), and those with more than one TMD (*n* = 6) (Table 3).

**Figure 2:**
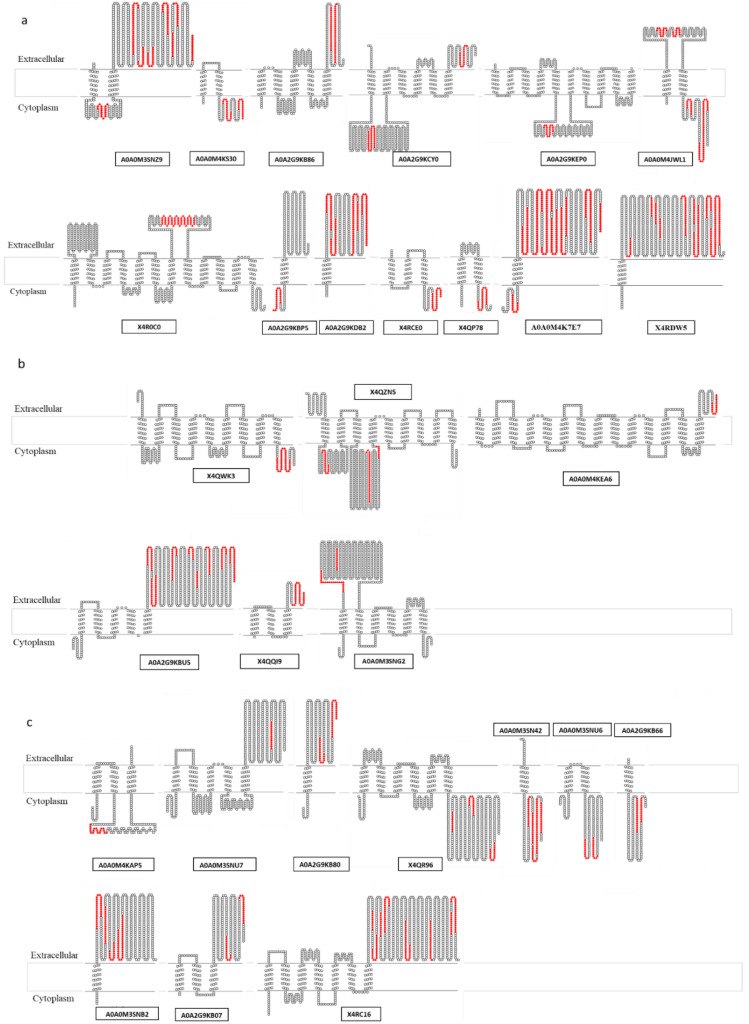
Topological representation of the identified membrane proteins in the “pan-surfome” analysis of the 15 *T. pyogenes* isolates. The TOPO2 Transmembrane Protein Display was used to perform the representation and the TMHMM algorithm to predict transmembrane domains (TMD). (**a**) Membrane proteins identified in more than 70% of the analysed strains. (**b**) Membrane proteins which were present in 50–70% of strains. (**c**) Membrane proteins identified in 30–50% of strains. In red are shown the peptides experimentally identified by LC-MS/MS.

Their topology was studied by means of TOPO2 after mapping the experimentally identified peptides (Appendix A, Supplementary Materials Dataset A1) on the predicted sequences. The membrane proteins included in the ranking were those with the majority of peptides oriented to the external side of the membrane (Figure 2), either with one or more than one TMD.

Another criterion to select proteins as antigen candidates for further studies was that they were highly conserved among the isolates. The degree of homology in the amino acid sequence of each protein was compared with the 10 sequenced *T. pyogenes* strains that are published so far. All the proteins listed in the ranking showed a degree of homology in their amino acid sequence that ranged from 82.5% to 100% among all the completely sequenced isolates of *T. pyogenes* (data not shown).

Finally, the algorithm VaxiJen was used to predict the protective capacity of those proteins included in the ranking. Most of the proteins which were previously included in the ranking reached 0.5 (ranged from 0.50 to 0.8727). Considering the average of the score per protein category (regardless of the group A, B or C), an average of 0.61 on the VaxiJen score was observed for cell wall proteins, secreted proteins and lipoproteins. A lower score was obtained for the membrane proteins, either with one TMD (0.55) or more than one TMD (0.55).

Some proteins were removed from the rating because their VaxiJen scores were lower than 0.5, and therefore they were considered as potential non-antigens: in the category A, the membrane protein X4RDW5, annotated as a signal peptidase I and present in all the clinical isolates, was removed from the rating because its VaxiJen score was 0.43. Within the category C, the cell wall anchored protein X4QMI5, the secreted protein A0A0M4JYB5, the lipoproteins A0A0M5KPJ2 and A0A2G9KEH2 and the membrane proteins A0A2G9KB80, X4QR96 and A0A0M3SNU6 (0.40; 0.48; 0.45; 0.46; 0.48, 0.48 and 0.47, respectively) were also removed. The majority of the excluded proteins (shown in bold in Table 3) belonged to the category of membrane proteins.

## 4. Discussion

Surface proteins play an essential role in the interplay between cells and their environment, which is even more relevant for microorganisms causing infectious diseases, as many of these proteins are involved in virulence or pathogenicity [11,27]. Moreover, surface proteins, as being exposed, have the highest chances to raise an effective immune response and, therefore, to become ideal candidates for drug or vaccine development [28].

Proteomics offers an adequate tool for massive identification of proteins. Particularly, the “shaving” of bacterial live cells with proteases followed by LC-MS/MS analysis constitutes a powerful technique for the fast identification of the most abundant and exposed surface proteins, i.e., the “surfome” [12,19,29]. In the present study, we performed, for the first time, the “shaving” approach to identify the “pan-surfome” of *T. pyogenes* and to carry out a comparative analysis of the surface protein profile among several clinical isolates. A total of 140 surface proteins were identified, similar to that obtained for other pathogens [12,19,30,31]. It demonstrates the utility of the proteomic “shaving” of live cells for the detection of surface proteins and to describe the “pan-surfome” of *T. pyogenes*. As expected from previous applications of this proteomic approach, we also found a substantial number of cytoplasmic proteins. It is widely known that the identification of predicted cytoplasmic proteins in bacterial surface fractions is not strange and an ineluctable fact. It can be due to several reasons, as residual cell lysis, export by non-canonical secretion pathways (i.e., “moonlighting” proteins) or release via extracellular membrane vesicles [32].

When we compared the expression frequencies of the different categories in relative terms, the secreted and the cell wall proteins were those most prevalent, as those two categories exhibited the highest number of proteins identified in a high proportion of isolates (six out of 10 secreted proteins and eight out of 25 cell-wall proteins in ≥50% of the isolates, respectively). This indicates that those protein categories are most exposed on the surface of Gram-positive bacteria, as already reported [11]. Very similar results have been obtained by proteomic analysis in other Gram-positive species, such as *Streptococcus pneumoniae, Streptococcus suis, Enterococcus faecalis* and group A *Streptococcus* [12,19,30,31].

Noticeably, we identified 11 predicted cell wall proteins that possessed an LAXTG motif instead of the LPXTG that is the most common in Gram-positive bacteria. In addition, 10 out of the 25 identified cell wall proteins had an LSXTG motif. Additionally, we identified the membrane protein sortase E. This class of sortases has been recently described in bacteria with high G + C content [33,34]. Sortase E would act as the housekeeping sorting enzyme in *T. pyogenes*, as sortases A and E are never found in the same organism. Sortases E recognize substrates containing the LAXTG motif [35,36]. However, there is no published evidence that they can act on LSXTG, although it cannot be discarded.

Considering that intra-species genetic variability can take place and that the protein expression pattern among isolates varies, finding a lot of proteins that are common to the majority of the analyzed isolates would not be expected [12,19,37]. In fact, in *Streptococcus pneumoniae* it has been reported that only 10.5% of the identified surface proteins were common to all the analyzed isolates [12] and in *Streptococcus suis,* Gómez-Gascón et al., did not identify any common protein in the 100% of the tested strains (*n* = 39) [19]. In this study, 51 surface proteins were identified in more than 50% of the strains. This also indicates a variability of protein expression pattern among all the isolates, as shown by the PCA. In addition, our hierarchically-clustered heatmap analysis showed that lipoproteins, cell wall proteins and membrane proteins to a lesser extent contributed to these differences among the clinical isolates, in a similar way to a recent study in *S. suis* [38]. Moreover, when we searched for a relationship between the protein pattern expression and the pulsed field gel electrophoresis (PFGE) clusters in our *T. pyogenes* isolates, no significant correlation was obtained (data not shown). Those findings coincide with the results showed previously by our group, i.e., a relatively high genetic diversity in this bacterial species [4]. We do not know whether there is a correlation or not between these differences and other biological features, such as antimicrobial susceptibility/resistance patterns.

After describing the “pan-surfome”, we ranked the identified proteins in three different groups (A, B, C; from best to worst) based on three criteria: to be (i) surface-exposed, (ii) highly conserved and (iii) widely distributed among different isolates. Following these criteria, 16 proteins were included in the group A, nine proteins were included in the group B, and 15 proteins were included in the group C. We assumed that proteins belonging to the categories of cell-wall anchored, secreted and lipoproteins are highly accessible to antibodies if they were identified with this proteomic procedure, as already demonstrated [7], and must be considered as the best option for further studies. Therefore, we included two, two and six cell-wall proteins in the groups A, B and C, respectively. For the lipoprotein category, three were included in the groups A and C and 2 were ranked in the group B. Regarding secreted proteins, four, two and one were included in the groups A, B and C, respectively.

Although several membrane proteins could be included in any group, just seven, three and five proteins were included in the groups A, B and C, respectively. Membrane proteins are, in principle, more embedded and therefore less surface-exposed and accessible, unless they have domains large enough to reach the surface through the peptidoglycan layer. In addition, for some of these membrane proteins the identified peptides matched loops that are theoretically predicted to be in intracellular domains. This can be due either to misleading predictions by subcellular localization/topology algorithms [12,19,30,39], or to release of such domains because of residual lysis. These facts make membrane proteins, a priori, worse candidates than cell wall-anchored or lipoproteins.

Finally, we used the algorithm VaxiJen, which is based on the physico-chemical properties of proteins, to predict the protective capacity of those proteins included in the ranking categories. According to the average score obtained in VaxiJen for cell wall proteins, secreted proteins and lipoproteins (score = 0.61) would be considered the best antigens to raise a high and effective immune response, in comparison with membrane proteins possessing one TMD (0.55) and membrane proteins with more than one TMD (0.55). Those findings agree with the statement published by other authors that cell wall-anchored proteins, lipoproteins and secreted proteins are the best options to this purpose [10,11]. Moreover, most of the proteins that were previously included in the ranking reached the threshold value of 0.5.

Some proteins belonging to different categories were removed from the ranking as they had a VaxiJen score lower than 0.5 and were classified as non-antigens. Most of the excluded proteins belonged to the category of membrane proteins.

According to our criteria, a total of 16 proteins (two cell wall proteins, three lipoproteins, four secreted proteins and seven membrane proteins) fulfilled requirements to be appropriate candidates for further immunization and vaccine studies. All of them were widely distributed (present in ≥70% of isolates) and highly conserved.

It should be noted that pyolysin (Q9S0W7) was rated in the best group of our ranking, as it was identified in 100% of the analyzed isolates. This reveals that this protein is a good putative candidate to be considered in further studies to develop a vaccine or for being included in new diagnostic tools. This is not surprising because it is considered the major virulence factor of *T. pyogenes* encoded by the gene *plo*, which has been detected in all wild-type strains described until now [2]. Jost et al., in 2003, tested a vaccine based on PLO with the detection of specific antibodies in sera of immunized mice and showing protection against infection [40]. Other authors have developed a vaccine against multiple pathogens, *T. pyogenes* and *C. perfringens*, with really promising results [17,41]. However, different authors have questioned that the results obtained in murine models can be applied to pigs [42]. Nonetheless, the current tendency is genetic immunization, like the vaccine developed by Huang et al. in 2018 that consisted of a DNA vaccine containing genes encoding four different *T. pyogenes* virulence factors [43].

This work provides interesting information on the field of proteomics applied to the control of infectious diseases, since it makes it possible to find surface antigens for the development of new diagnostic tools and recombinant subunit vaccines against *T. pyogenes*, a not well known pathogen.

## 5. Conclusions

The proteomic “shaving” of live cells is a useful tool for the detection of common proteins to describe the “pan-surfome” of *Trueperella pyogenes* Moreover, two cell wall proteins (X4QWN2, X4R8M3), three lipoproteins (A0A0M3SNR1, A0A0M4K9G4, A0A0M4JY33), four secreted proteins (X4R0V4, A0A2G9KEL5, Q9S0W7, X4QUK6) and seven membrane proteins (A0A0M4K7E7, A0A2G9KDB2 A0A0M3SNZ9, A0A0M4KS30, A0A2G9KB86, A0A2G9KCY0, A0A0M4JWL1) were identified in more than 70% of the studied isolates, were highly expressed and highly conserved. These proteins could be good putative antigen candidates, alone or in combination, in future vaccination studies, or for being included in appropriate tools to diagnose infections caused by this pathogen.

## Figures and Tables

**Table 1:** *Trueperella pyogenes* isolates (*n* = 15) obtained from pigs analysed in this study.

Proteomics Reference	Origin ^a^	Rearing System ^b^	PFGE Pattern ^c^	Cluster ^d^
A	Liver	Extensive	43	B
B	Lung	Intensive	28	A
C	Lymph nodes	Intensive	43	B
D	Brain	Intensive	53	C
E	Lung	Intensive	37	B
F	Joint	Intensive	37	B
G	Lymph nodes	Extensive	12	A
H	Lymph nodes	Extensive	1	A
I	Liver	Extensive	12	A
J	Spleen	Extensive	44	B
K	Lymph nodes	Extensive	43	B
L	Heart	Intensive	43	B
M	Joint	Intensive	13	A
N	Heart	Intensive	44	B
O	Abscess	Extensive	64	C

^a^ All the isolates were recovered from pigs totally or partially condemned at the slaughterhouse after veterinary inspection (Regulation 2004/854/EC). Samples were obtained from lymph node (*n* = 4), lung (*n* = 2), joint (*n* = 2), liver (*n* = 2), heart (*n* = 2), spleen (*n* = 1), abscess (*n* = 1), brain (*n* = 1), with macroscopic lesions of pneumonia, endocarditis, arthritis, lymphadenitis, abscess or pyogranuloma-like lesions. ^b^ Strains were isolated from carrier pigs which were reared under intensive or extensive farming conditions. ^c^ Pulsed field gel electrophoresis (PFGE) patterns obtained after macrorestriction with the BcuI enzyme showing the genetic relationship between *T.pyogenes* isolates [4]. ^d^ All the isolates analysed in the previous study were grouped within three main PFGE clusters at an 85% of genetic similarity (A–C) [4].

**Table 2:** Summary of identified surface proteins in *Trueperella pyogenes* isolates by “shaving” cells and liquid chromatography-mass spectrometry (LC-MS/MS) analysis.

Protein Category ^a^	Identified Proteins among All the Strains	Range of Identified Proteins Per Strain
Surface proteins	140	35–73
Lipoprotein	23 (16.4%)	1–13
Cell Wall	25 (17.9%)	13–25
Secretory	10 (7.1%)	5–10
Membrane	82 (58.6%)	22–50

^a^ Protein categories based on subcellular localization were established according to PsortB v3.0. Other algorithms were also used to contrast those predictions: TMHMM 2.0 for searching transmembrane helices; SignalP 3.0 for type-I signal peptides which are present in secreted proteins; LipoP for identifying type-II signal peptides, which are characteristic of lipoproteins. Cell wall proteins were those having a sortase recognizing motif. The proteins that presented doubtful location were manually inspected.

**Table 3:** Rating of proteins according to their potentiality as putative antigens for further immunization and/or vaccination studies.

Ranking	Proteins
A (16) ^a^	Cell wall: X4QWN2, X4R8M3 Lipoproteins: A0A0M3SNR1, A0A0M4K9G4, A0A0M4JY33 Secreted proteins: X4R0V4, A0A2G9KEL5, Q9S0W7, X4QUK6 Membrane proteins (1 TMD): A0A0M4K7E7, A0A2G9KDB2, **X4RDW5** Membrane proteins (>1 TMD): A0A0M3SNZ9, A0A0M4KS30, A0A2G9KB86, A0A2G9KCY0, A0A0M4JWL1
B (9)	Cell wall: A0A2G9KC18, A0A2G9KBF5 Lipoproteins: A0A0M5KH79, A0A0M4KR83 Secreted proteins: A0A2G9KD79, A0A2G9KA87 Membrane proteins (>1 TMD): X4QZN5, A0A2G9KBU5, A0A0M3SNG2
C (15)	Cell wall: X4RE32, X4RB57, A0A0M5KIG4, X4RCL8, **X4QMI5**, A0A2G9KAD4, A0A0M4K5P6 Lipoproteins: **A0A0M5KPJ2**, A0A0M4K9R6, X4QXA0, **A0A2G9KEH2**, A0A0M4K5B8 Secreted proteins: A0A0M4KB23, **A0A0M4JYB5** Membrane proteins (1 TMD): **A0A2G9KB80**, A0A0M3SN42, A0A2G9KB66, A0A0M3SNB2 Membrane proteins (>1 TMD): **X4QR96**, **A0A0M3SNU6**, A0A2G9KB07, X4RC16

^a^ A: >70% analyzed serotypes; B: 50–70% analyzed serotypes; C: 30–50% analyzed serotypes. We classified the membrane proteins in two different groups, membrane proteins with one transmembrane domain (TMD) and membrane proteins with more than one transmembrane domain. The proteins which were excluded because they did not reach a VaxiJen score ≥0.5 are highlighted in bold.

## Supplementary Materials

The following are available online at https://www.mdpi.com/2076-393X/8/2/314/s1. Supplementary Table 1: List and quantitative values of identified surface proteins, as predicted by PsortB v3.0, Supplementary Figure S1: Principal component analysis (PCA) of global surface proteins identified in the 15 *Trueperella pyogenes* clinical isolates, Supplementary Figure S2: Euclidean distances among biological replicates of global surface proteins identified in the 15 *Trueperella pyogenes* clinical isolates.

## Appendix A

The complete lists of proteins, peptides, and all raw data information for the each of the three replicates of the fifteen clinical isolates, including chromatography peak areas for further quantification, are available as supplementary material in this work as Supplementary Materials Dataset A1.
