# Supplementary figures and images for: Search of Potential Vaccine Candidates against Trueperella pyogenes Infections through Proteomic and Bioinformatic Analysis
Source: Vaccines (Basel). 2020 Jun 17;8(2):314. doi: 10.3390/vaccines8020314 (PMC7350218; doi:10.3390/vaccines8020314)

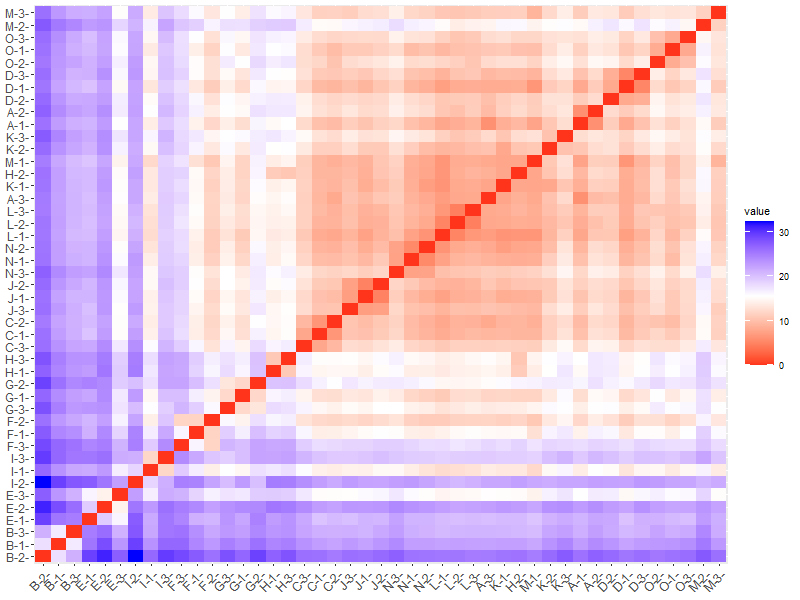

Supplement: Supplementary file 1 [file vaccines-08-00314-s001.zip › Supplementary Figure 2.tif]
